# Supplementary material for: Decomposition of a set of distributions in extended exponential family form for distinguishing multiple oligo-dimensional marker expression profiles of single-cell populations and visualizing their dynamics
Source: PLoS One. 2020 Apr 10;15(4):e0231250. doi: 10.1371/journal.pone.0231250 (PMC7147751; doi:10.1371/journal.pone.0231250)
Supplement: S1 Text — (PDF) [file pone.0231250.s001.pdf]

Supplementary Text for DEEF

Daigo Okada and Ryo Yamada

February 10, 2020

1 Introduction to information geometry and exponential families

Research on information geometry has focused on exponential families and the coordinate space of probability distributions. An exponential family is a probability distribution that can be expressed in the following form:

$$\log P(x|\theta) = C(x) + \sum_{i=1} F_i(x)\theta_i - \psi(\theta)$$

where $P(x|\theta)$ is the probability density function, $C(x)$ is a function of x only, θ is the scalar value vector given for each distribution, θ_i is the i-th element of θ, $F_i(x)$ is the coefficient function of θ_i, and $\psi(\theta)$ is a potential function such that $P(x|\theta)$ satisfies the definition of a probability density distribution. Many probability distributions, including the standard normal distribution, can be expressed in this form and are included in the exponential family. If a distribution can be expressed as an exponential family, θ coordinates can be applied to it and it can be embedded in a low-dimensional space, which is a statistical manifold [1]. This space has two flat coordinate systems on which KL divergence can be calculated using each coordinate value [2]. In addition, the relation between the probability density/mass functions of θ coordinates is defined by $F_i(x)$. The mathematical nature of this space is well known in information geometry. However, some distributions are not included in the exponential family, such as the mixture normal distribution, which is commonly used in biology.

References

[1] Amari, S. I. "Information geometry." Contemporary Mathematics 203 (1997): 81-96.

[2] Nielsen, Frank, and Richard Nock. "Entropies and cross-entropies of exponential families." 2010 IEEE International Conference on Image Processing. IEEE, 2010.

2 Simulation analysis implementation

2.1 Embedding into θ coordinate space

First, we applied DEEF to a set of instances of the distribution in the exponential family and to a set of instances that are a parametric mixture of distributions in the exponential family to validate our theory. We generated four sets of simulation instances of a distribution using the monovariate normal distribution. The four sets are denoted 2D, Random, 1D, and Mixture. 2D consisted of 900 instances of a normal distribution, with the mean ranging from -1 to 1 and the sd ranging from 2 to 4 at a fixed interval of 0.069 for each. Random consisted of 50 instances randomly sampled from 2D. 1D was a normal distribution set that made a one-dimensional manifold in the same space as that of 2D. Mixture consisted of 900 instances that were a mixture of two normal distributions; one normal distribution was N(-1,1) and the other distribution had mean and sd ranging from 4 to 5 and 2 to 4 at fixed intervals of 0.034 and 0.069, respectively. The mixture ratio of the two distributions was 0.5 for all instances (Fig A). The number of grids was 10,000. The range for discretization was determined so that the section between the 0.5th percentile and the 99.5th percentile of all distributions was included.DEEF successfully extracted the parameter structure and reconstructed the distributions. The θ coordinate were calculated using the theoretical value of the functional inner product defined by the mean and sd.

The results of the application of our method to these four sets are shown in Fig A. The eigenvalues corresponding to each θ coordinate are shown in Fig B. For all distribution sets, the maximum eigenvalue is negative. The θ coordinate is denoted θ_i in decreasing order of eigenvalues.

θ_{last} is the coordinate corresponding to the lowest eigenvalue whose absolute value is largest. The eigenvalues calculated using this method always contain negative values (details given in the Appendix). Only the top two or three positive eigenvalues have meaningful contributions; the other positive eigenvalues have essentially no contribution. The number of parameters used to describe the heterogeneity of instances, or DoFs, for 2D is 2 (mean and sd). The DoFs for Random, 1D, and Mixture are 2, 1, and 2, respectively. These numbers correspond to the numbers of positive eigenvalues with meaningful absolute values.

We embedded all instance distributions into a three-dimensional space with the top three absolute eigenvalues (third column in Fig A). In the θ coordinate space, the original parameter structure, indicated by the color pattern, was maintained for all four sets. It can also be seen that the distributions were embedded on the manifold with the dimension of the original parameter structure.

Next, we investigated the $C(x)$ and $F(x)$ of the top coordinates of each distribution set. Fig C shows the calculated $C(x)$, $F_{last}(x)$, $F_1(x)$, and $F_2(x)$ for 2D, Random, 1D, and Mixture. For distribution set 2D, $C(x)$ has information about the average feature of the whole distribution set, as shown by the black curve (Fig C(a)). This function is convex, with a peak at the center of the x coordinate, which is the average pattern in distribution set 2D. θ_1 and θ_2 are

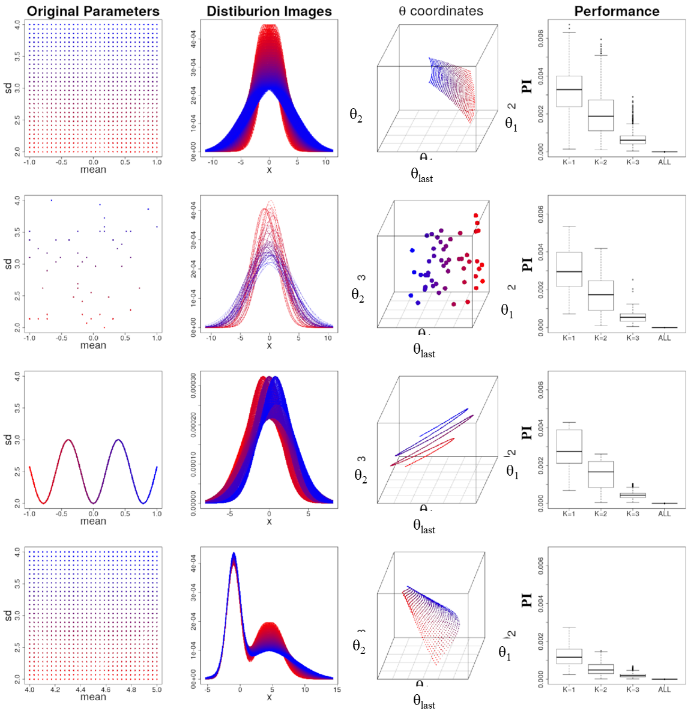

Fig A. Original parameter structure (first column), distribution (second column), θ coordinate mapping (third column), and boxplots of performance (fourth column) for four types of distribution set (2D, Random, 1D, Mixture). Each dot in the first and third column panels and each line in the second column panels together represent a distribution. Embedding in the θ coordinate space reproduces the original parameter structure with distortion. The fourth column panels show boxplots of the Performance Index (PI) defiled by the sum of the squared error of distributions reconstructed using only the top K coordinates with high absolute eigenvalues for each distribution set. As K increases, the reconstructed distribution set approaches the original distribution set. When all θ coordinates are used, all distributions belonging to the reconstructed distribution set are identical to the original distributions.

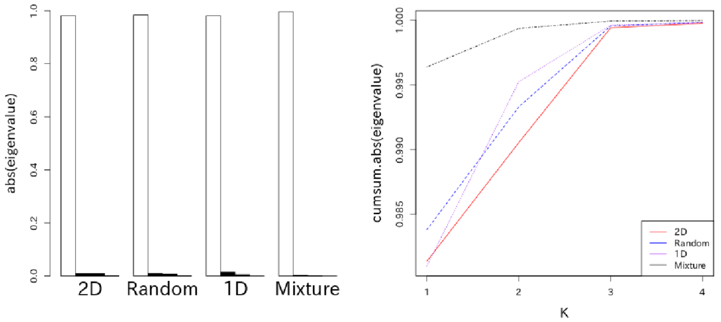

Fig B Eigenvalue plots for each distribution set. The left panel shows the absolute eigenvalues standardized so that its total value was 1, where the black bars are positive eigenvalues and the white bars are negative eigenvalues. For all distribution sets, the maximum eigenvalue was negative. The right panel shows the cumulative sum of eigenvalues. In distribution set 2D, the contribution increases by θ_1 and θ_2 are almost equal. This corresponds to a degree of freedom (DoF) of 2 for the parameter structure. This tendency also appears for Random and Mixture, although θ_1 has greater explanatory power. For distribution set 1D, the contribution of θ_2 is greatly reduced compared to that of θ_1.

the coordinates corresponding to the positive eigenvalues. $F_1(x)$ indicates that a larger value of θ_1 leads to a larger probability mass at both ends (Fig C(a), blue line). This is consistent with the fact that the distributions Normal(-1, 4) and Normal(1, 4) were embedded into the region with the largest θ_1 coordinate value in Fig A. $F_2(x)$ indicates that a larger value of θ_2 leads to a larger probability mass at the right end and a smaller probability mass at the left end (Fig C(a), purple line). The distribution with the maximum θ_2 value is Normal(1, 2), which is the member distribution with the highest mean and the lowest sd value in distribution set 2D in Fig A. These results suggest that $F_i(x)$, which corresponds to the positive eigenvalues, has information about what part of the difference each θ coordinate explains in the original distribution. $F_{last}(x)$ suggests that θ_{last}, the coordinate with the largest negative eigenvalue, is almost parallel to the x-axis and has little information about the distribution feature (Fig C(a), red line). This axis distorts the inner products and distances between the points on the manifold. For distribution set Random, a similar result was obtained but with slight distortion (Fig C(b)). Interestingly, the $F_1(x)$ and $F_2(x)$ for distribution set 1D are similar to $F_2(x)$ and inverted $F_1(x)$, respectively (Fig C(c)). For distribution set Mixture, extremely large values tended to be estimated in the edge region (Fig C (d)). Mixture(sub) is the magnified view of the central part of Mixture and shows that $C(x)$ captures the bimodality of the mixture normal distribution. Each $F_i(x)$ has a unique complex pattern in the distribution set, as is the case for the normal distribution set.

Finally, we reconstructed the distribution set using the top θ coordinates and

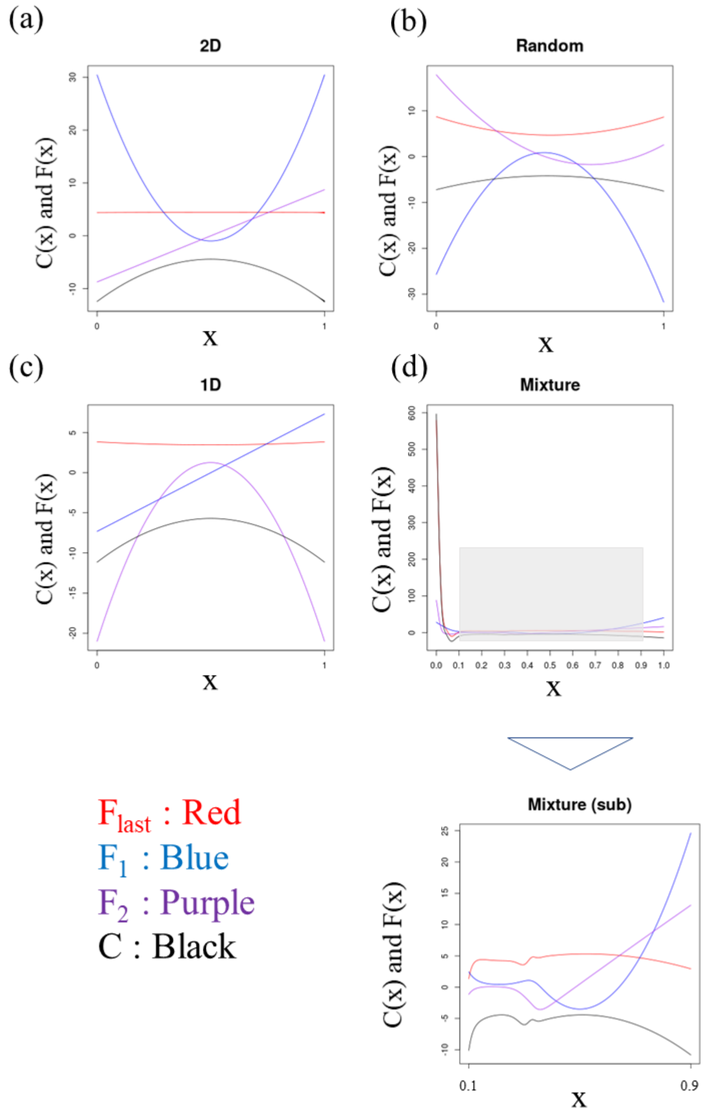

Fig C. Calculated $C(x)$, $F_{last}(x)$, $F_1(x)$, and $F_2(x)$ for distribution sets 2D, Random, 1D, and Mixture. For Mixture, extreme values tend to be estimated in the edge region. The range of discretization is scaled from 0 to 1. Part of the plot between 0.1 and 0.9 of the entire region (indicated by gray rectangle) was extracted from the left panel (Mixture(sub)). $C(x)$ represents the average pattern of the distribution set. $F_i(x)$ is a function that associates the corresponding θ_i with the original distribution.

evaluated performance. The reconstruction performance is defined as the difference between the original distribution and the reconstructed distribution (details are given in the main manuscript). With an increasing number of coordinates, the reconstructed distribution tends to approach the original distribution as a whole (fourth column in Fig A). Using all coordinates, the original distribution can be exactly reproduced. An example of the reconstruction of one distribution is shown in Fig D(a). Fig E shows the relationship between the position on the original parameter coordinates and performance. The performance improved as the number of coordinates increased to three. Instances located at the periphery of the distribution set tended to have worse reconstruction performance and required more θ coordinates to achieve performance similar to that of instances in the central area. The features shared by many instances were explained by a limited number of coordinates with relatively large eigenvalues, whereas those of instances at the periphery required more coordinates with relatively small eigenvalues. For example, Normal(-1, 4) is not sufficiently reproduced at the edge for K=3 or 4; K=5 is required (Fig D(b)). These performance features apply to all four distribution sets. These evaluations indicate that our method can identify the EEF expression of a set of distributions in the exponential family and can be applied to a set of mixture distributions that are not in the exponential family.

As another case of applying DEEF to simulation data, Fig F shows the case of an exponential distribution set. An exponential distribution was parameterized by one parameter and a distribution with non-symmetric shape that was unlike a normal distribution. We generated an exponential distribution set as another example of the application of DEEF. This set consisted of 900 instances whose lambda ranged from 1 to 5 at an interval of 0.0044. The number of grids was 10,000. The range for discretization was determined so that the section between the 0.5th percentile and the 99.5th percentile of all distributions was included. DEEF successfully extracted the parameter structure and reconstructed the distributions. Before applying DEEF, the first grid was removed for the calculation. Interestingly, the maximum eigenvalue was a positive eigenvalue, unlike the case for the normal distribution set. However, at least θ_1 and θ_{last} (K=2) are needed to obtain good performance in reconstruction.

2.2 Relationship between complexity and number of eigenvalues

The eigenvalue plots of distribution sets 2D, Random, 1D, and Mixture imply that the number of significant positive eigenvalues is the DoF of the set of distributions. As mentioned, the DoFs of the original parameter structures of 2D, Random, 1D, and Mixture were 2, 2, 1, and 2, respectively, which correspond to the numbers of significant positive eigenvalues. We thus quantitatively investigated whether the potential DoF of the distribution can be estimated using the DEEF method for other mixture normal distribution sets. The number of mixture components was changed from 2 to 10. All component normal distributions had the same sd (=1). The mean values of the component normal distributions

(a)

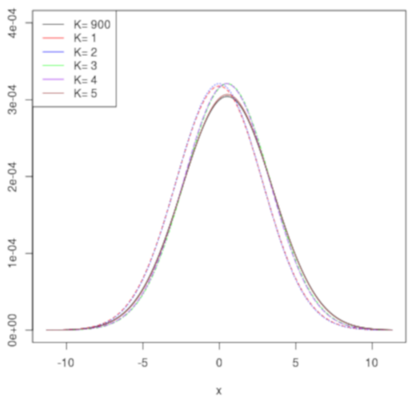

(b)

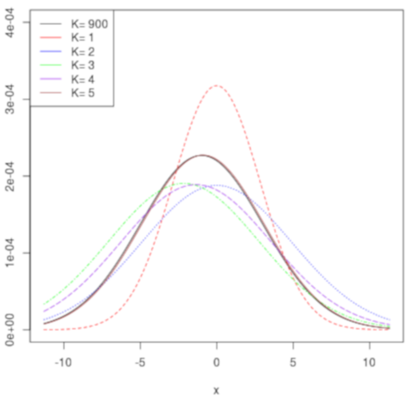

Fig D. Change in reconstructed distribution with K value (1, 2, 3, 4, 5, and all 900). (a) Normal(0.517, 2.97). The average and dispersion are roughly reproduced at K=3. The distribution reproduced with K=5 is almost equal to the original distribution (K=900). (b) Normal(-1, 4). The difference between this distribution and other distributions can be explained by θ_3 or θ_4, but not θ_1 or θ_2.

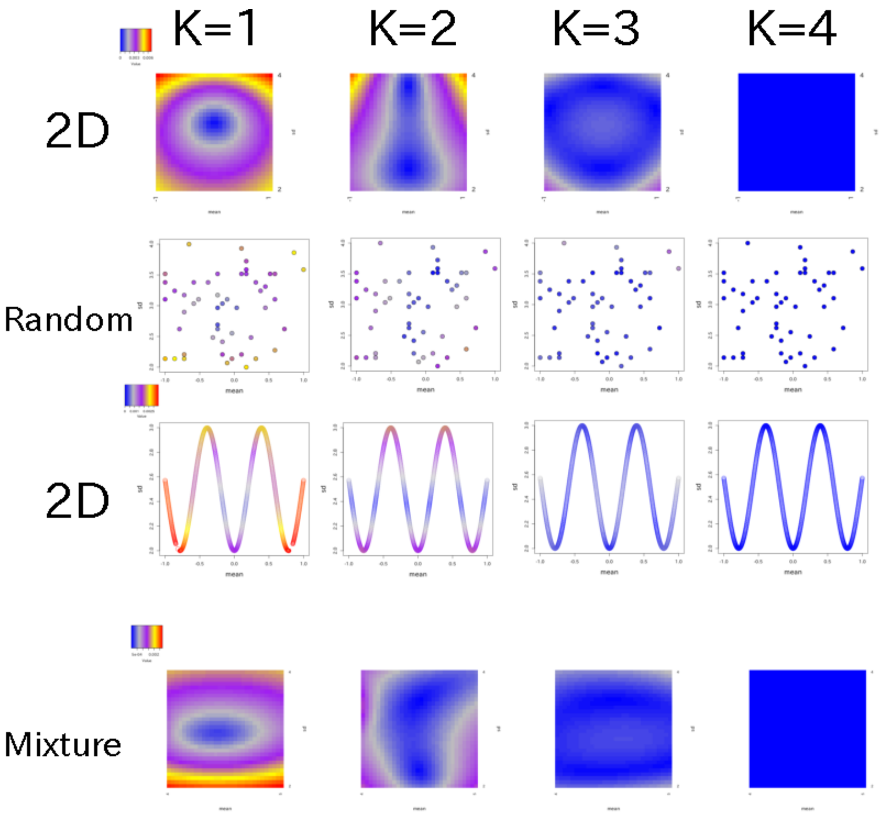

Fig E. Heat map of the performance of distributions reconstructed using only the top K coordinates for distribution sets 2D, 1D, Random, and Mixture. The performance, indicated by color, was evaluated in terms of the squared error between the true probability mass function and the probability mass function reconstructed using the top K θ coordinates. These panels show the relation between the location on the original parameter structure and the reconstruction performance.

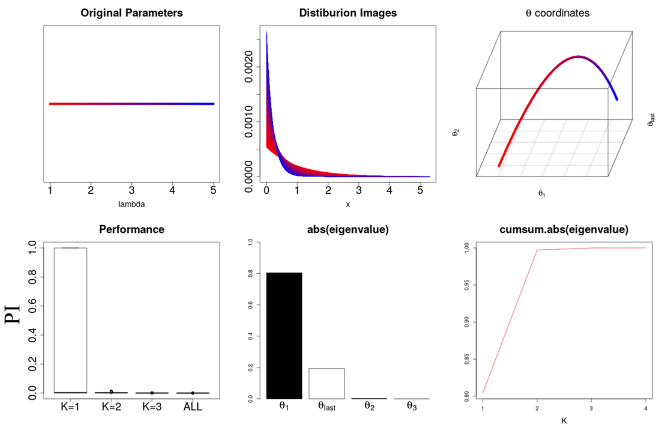

Fig F. Application of DEEF to an exponential distribution set. The meanings of the panels are the same as those for the normal distribution set.

were evenly spaced between -10 and 10. The number of non-negligible positive eigenvalues was defined as the minimum number of eigenvalues whose sum exceeds 90% of the sum of all positive eigenvalues. A distribution set was composed of 100 distributions, among which only the mixture ratio was different. For this case, the potential DoF for the distribution set was defined as the number of components - 1 because only the mixture ratio varied.

Fig G shows a plot of the potential DoF and the number of non-negligible positive eigenvalues. This plot suggests that the potential DoF corresponds to the number of non-negligible positive eigenvalues when the number of components is small (red line). When the potential DoF was larger, the number of required eigenvalues decreased, which seemed to be due to the insufficient resolution of the decomposition by the proposed method because of the relatively small sample size compared to the complexity of the datasets. This result suggests that our method can theoretically identify the potential DoF of datasets based on the quantity of meaningful information in the datasets.

2.3 Discussion in terms of information geometry

In information geometry, the geometric properties of the probability distribution space have been extensively researched. In particular, it is known that exponential families can be embedded into special manifolds equipped with two flat coordinate systems. The geometrical properties of these coordinate systems have been well investigated. The θ coordinate system of the DEEF method is quite similar to such systems. However, the potential function of an EEF is not convex and has imaginary axis (particularly when the inner product matrix is calculated from the probability mass function, a negative eigenvalue must be appear. (proof is shown in Appendix Theorem 3)). That makes the interpretation of the EEF space more complicated than that of a regular information geome-

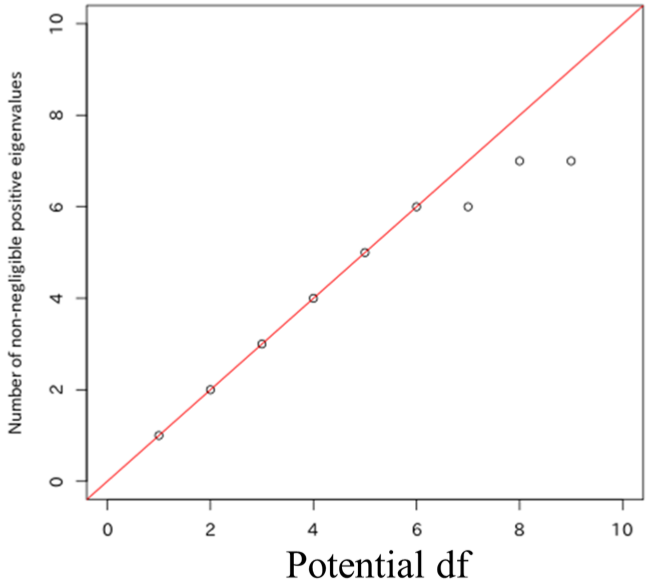

Fig G. Plot of potential degrees of freedom (DoFs) versus the number of non-negligible positive eigenvalues. The number of mixture components was varied from 2 to 10. The potential DoF can be defined as the number of mixture components - 1. The number of non-negligible positive eigenvalues is equal to the potential DoF of the distribution set; however, the relationship is not satisfied for large DoFs due to the limited resolution of the proposed method.

try space. One specific feature of the EEF space is that it has subspaces where no distributions are assigned. One example of a space with an indeterminate inner product is the Minkowski space, which has a deep relationship with special relativity [1]. The properties of manifolds defined by these features should be further studied in the future. The investigation of these features in terms of theoretical geometry would further advance the understanding of statistical manifolds and probability distribution theory.

References

[1] Walter, Scott. "The non-Euclidean style of Minkowskian relativity." The Symbolic Universe, Editor J. Gray, Oxford University Press, Oxford (1999): 91-127.

Appendix

Theorem 1 If $P(x, \boldsymbol{\theta}^P)$ and $Q(x, \boldsymbol{\theta}^Q)$ are the members of an exponential family represented by Eq 1, then:

$$< P(x, \boldsymbol{\theta}^P), Q(x, \boldsymbol{\theta}^Q) >= \frac{e^{\psi(\boldsymbol{\theta}^P + \boldsymbol{\theta}^Q)}}{e^{\psi(\boldsymbol{\theta}^P)} e^{\psi(\boldsymbol{\theta}^Q)}}$$

Proof: The definition of an exponential family can be written as:

$$\log P(x, \boldsymbol{\theta}^P) = \sum_{i=0} F_i(x) \boldsymbol{\theta}_i^P - \psi(\boldsymbol{\theta}^P)$$
$$\log Q(x, \boldsymbol{\theta}^Q) = \sum_{i=0} F_i(x) \boldsymbol{\theta}_i^Q - \psi(\boldsymbol{\theta}^Q)$$
$$F_0(x) = C(x), \theta_0 = Const$$

Then, the inner product of the exponential distribution family is defined by the following procedure:

$$\begin{aligned}
< P(x, \boldsymbol{\theta}^P), P(x, \boldsymbol{\theta}^Q) > &= \int P(x, \boldsymbol{\theta}^P) P(x, \boldsymbol{\theta}^Q) dx \\
&= \int e^{\sum_{i=0} F_i(x) \theta_i^P - \psi(\boldsymbol{\theta}^P)} e^{\sum_{i=0} F_i(x) \theta_i^Q - \psi(\boldsymbol{\theta}^Q)} dx \\
&= \int e^{\sum_{i=0} F_i(x)(\theta_i^P + \theta_i^Q) - (\psi(\boldsymbol{\theta}^P) + \psi(\boldsymbol{\theta}^Q))} dx \\
&= \frac{1}{e^{\psi(\boldsymbol{\theta}^P)} e^{\psi(\boldsymbol{\theta}^Q)}} \int e^{\sum_{i=0} F_i(x)(\theta_i^P + \theta_i^Q)} dx
\end{aligned}$$

Let $\boldsymbol{\theta}^{P+Q}$ be $\boldsymbol{\theta}^P + \boldsymbol{\theta}^Q$. The above equation can then be rewritten as:

$$< P(x, \boldsymbol{\theta}^P), P(x, \boldsymbol{\theta}^Q) >= \frac{1}{e^{\psi(\boldsymbol{\theta}^P)} e^{\psi(\boldsymbol{\theta}^Q)}} \int e^{\sum_{i=0} F_i(x) \boldsymbol{\theta}^{P+Q}} dx$$

The following equation holds because: $\int P(x, \boldsymbol{\theta}^{P+Q}) dx = \int e^{\sum_{i=0} F_i(x) \theta_i^{P+Q} - \psi(\boldsymbol{\theta}^{P+Q})} dx = 1$

$$\int e^{\sum_{i=0} F_i(x) \theta_i^{P+Q}} dx = e^{\psi(\boldsymbol{\theta}^{P+Q})}$$

Then, the inner product between members of an exponential family is expressed as:

$$
\begin{aligned}
< P(x,\boldsymbol{\theta}^P), P(x,\boldsymbol{\theta}^Q) > &= \frac{1}{e^{\psi(\boldsymbol{\theta}^P)}e^{\psi(\boldsymbol{\theta}^Q)}} \int e^{\sum_{i=0} F_i(x)\boldsymbol{\theta}^{P+Q}} dx \\
&= \frac{1}{e^{\psi(\boldsymbol{\theta}^P)}e^{\psi(\boldsymbol{\theta}^Q)}} \int e^{\sum_{i=0} F_i(x)\theta_i^{P+Q} - \psi(\boldsymbol{\theta}^{P+Q})} e^{\psi(\boldsymbol{\theta}^{P+Q})} dx \\
&= \frac{e^{\psi(\boldsymbol{\theta}^{P+Q})}}{e^{\psi(\boldsymbol{\theta}^P)}e^{\psi(\boldsymbol{\theta}^Q)}} \int e^{\sum_{i=0} F_i(x)\theta_i^{P+Q} - \psi(\boldsymbol{\theta}^{P+Q})} dx \\
&= \frac{e^{\psi(\boldsymbol{\theta}^{P+Q})}}{e^{\psi(\boldsymbol{\theta}^P)}e^{\psi(\boldsymbol{\theta}^Q)}} \\
&= \frac{e^{\psi(\boldsymbol{\theta}^P + \boldsymbol{\theta}^Q)}}{e^{\psi(\boldsymbol{\theta}^P)}e^{\psi(\boldsymbol{\theta}^Q)}}
\end{aligned}
$$

Theorem 2 If $P(x,\boldsymbol{\theta}^P)$ and $Q(x,\boldsymbol{\theta}^Q)$ are EEFs as defined in Eq 2, then:

$$
\frac{1}{2} \log < P(x,\boldsymbol{\theta}^P), Q(x,\boldsymbol{\theta}^Q) > = \sum_{k=1} h_k \theta_k^P \theta_k^Q
$$

Proof: If P and Q are not members of the exponential family but are EEFs, the potential function $\psi(\theta)$ can be expressed as $\psi'(\theta) = \sum_{k=1} h_k \theta_k^2$. Theorem 1 is also satisfied if P and Q are EEFs. Then:

$$
\begin{aligned}
\frac{1}{2} \log < P(x,\boldsymbol{\theta}^P), Q(x,\boldsymbol{\theta}^Q) > &= \frac{1}{2}(\psi(\boldsymbol{\theta}^{P+Q}) - \psi(\boldsymbol{\theta}^P) - \psi(\boldsymbol{\theta}^Q)) \\
&= \frac{1}{2}(\sum_{k=1} h_k(\theta_k^P + \theta_k^Q)^2 - \sum_{k=1} h_k(\theta_k^P)^2 - \sum_{k=1} h_k(\theta_k^Q)^2) \\
&= \sum_{k=1} h_k \theta_k^P \theta_k^Q
\end{aligned}
$$

Theorem 3 Matrix $\mathbf{M}$ must have at least one negative eigenvalue.

Proof: Probability matrix $\mathbf{P}$ can be expressed as:

$$
\mathbf{P} = \mathbf{N}\mathbf{A}
$$

where $\mathbf{A}$ has the same size as that of $\mathbf{P}$, $a_{i,j}$, the (i,j)-th element of $\mathbf{A}$, is non-negative, and $\mathbf{N}$ is a diagonal matrix used for the normalization of row sums whose i-th diagonal element is $N_i = \sum_{k=1}^m a_{i,k}$. Therefore, $\mathbf{Q} = \mathbf{P}\mathbf{P}^T = \mathbf{NA}(\mathbf{NA})^{\mathbf{T}} = \mathbf{NAA}^T\mathbf{N}$. Then, denote $q_{i,i}$ as the i-th diagonal element of $\mathbf{Q}$. $q_{i,i} = \frac{\sum_{k=1}^m a_{i,k}^2}{(\sum_{k=1}^m a_{i,k})^2}$ because $a_{i,j} > 0$ and $q_{i,i} < 1$. $trace(M) = \sum_{i=1}^n m_{i,i} = \sum_{i=1}^n \log q_{i,i}$, which must be negative. Because $\mathbf{M}$ is a symmetric matrix, it has n real eigenvalues. The trace and the sum of the eigenvalues must match. From the above, $\mathbf{M}$ has at least one negative eigenvalue.
