# Supplementary figures and images for: Decomposition of a set of distributions in extended exponential family form for distinguishing multiple oligo-dimensional marker expression profiles of single-cell populations and visualizing their dynamics
Source: PLoS One. 2020 Apr 10;15(4):e0231250. doi: 10.1371/journal.pone.0231250 (PMC7147751; doi:10.1371/journal.pone.0231250)

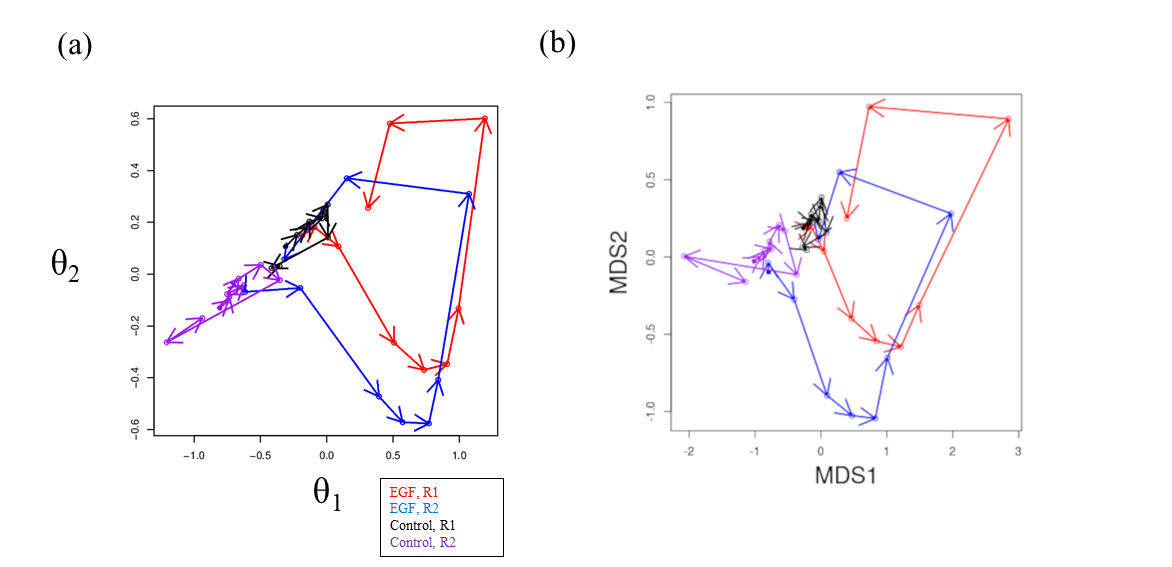

Supplement: S1 Fig — (a) θ coordinate plot for coordinates θ1 and θ2 and (b) MDS coordinate plot for two coordinates MDS1 and MDS2 with the top eigenvalues. (TIF) [file pone.0231250.s002.tif]

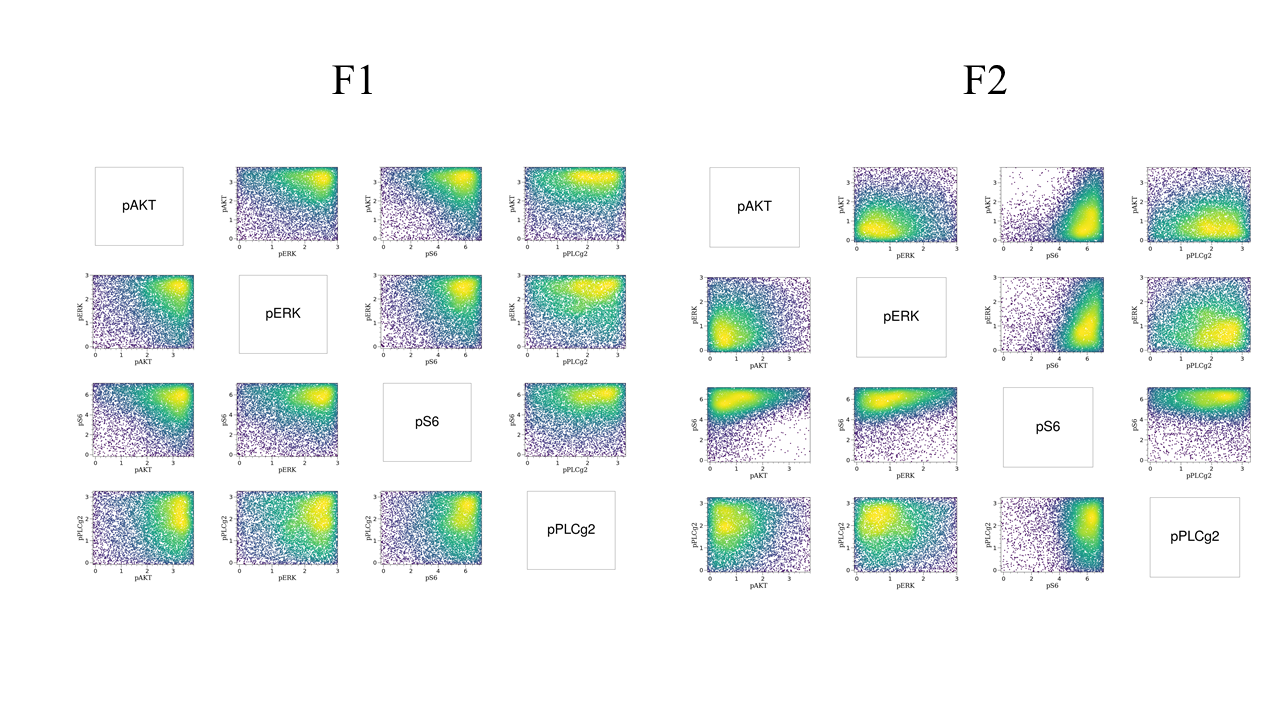

Supplement: S2 Fig — (TIF) [file pone.0231250.s003.tif]

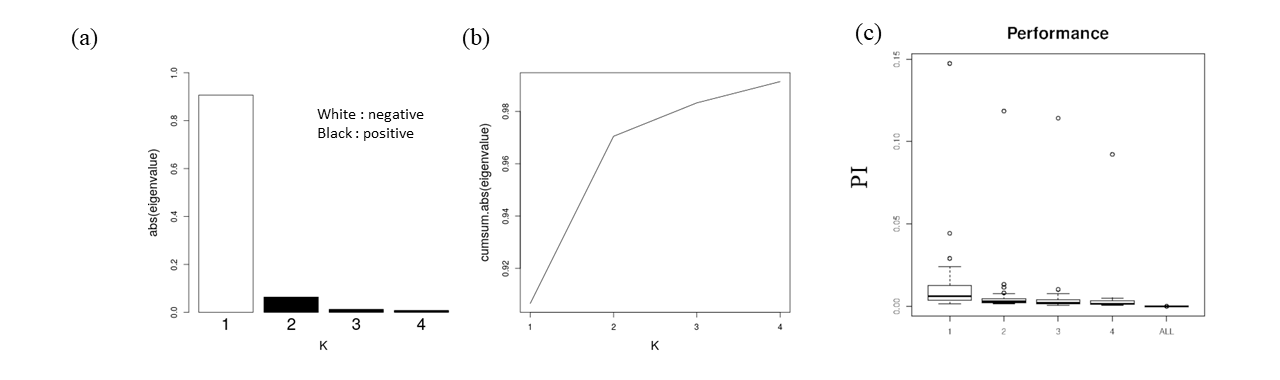

Supplement: S3 Fig — (a) Eigenvalue plots for an EGF stimulation dataset. Left panel shows the absolute eigenvalues standardized so that its total value was 1, where black bars are positive eigenvalues and white bars are negative eigenvalues. Right panel shows the cumulative sum of absolute eigenvalues. (b) Performance boxplot of distributions reconstructed using only the top K coordinates with high absolute eigenvalues for the EGF stimulation dataset. The performance was evaluated by the Performance Index (PI) defined by the sum of the squared error between the true probability mass function and the reconstructed probability mass function. The overall performance increases with increasing value of K. (TIF) [file pone.0231250.s004.tif]

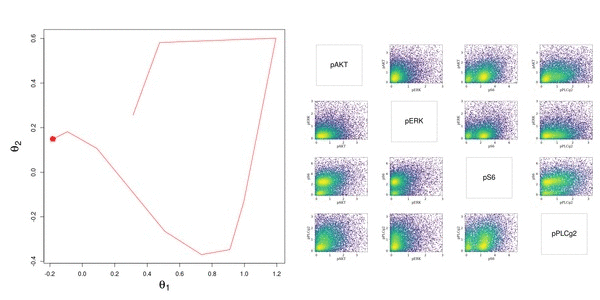

Supplement: S1 Movie — The reconstruction was done with θ1, θ2, and θlast (K = 3). (GIF) [file pone.0231250.s006.gif]
